# Seven-year trajectories of body weight, quality of life and comorbidities following Roux-en-Y gastric bypass and sleeve gastrectomy

**DOI:** 10.1038/s41366-021-01028-5

**Published:** 2022-01-01

**Authors:** Hans Jørgen Nielsen, Bjørn Gunnar Nedrebø, Alexander Fosså, John Roger Andersen, Jörg Assmus, Vigdis Halvorsen Dagsland, Simon Nitter Dankel, Oddrun Anita Gudbrandsen, Johan Fernø, Iren Hjellestad, Marianne Jensen Hjermstad, Ronette L. Kolotkin, Håvard Luong Thorsen, Gunnar Mellgren, Tone Nygaard Flølo

**Affiliations:** 1 Department of Surgery, Voss Hospital, Haukeland University Hospital, Voss, Norway; 2 Department of Medicine, Haugesund Hospital, Haugesund, Norway; 3 Department of Clinical Science, University of Bergen, Bergen, Norway; 4 Department of Oncology, Oslo University Hospital, Oslo, Norway; 5 KG Jebsen Centre for B-cell malignancies, Institute for Clinical Medicine, University of Oslo, Oslo, Norway; 6 Centre of Health Research, Førde Hospital Trust, Førde, Norway; 7 Department of Health and Caring Sciences, Western Norway University of Applied Sciences, Bergen, Norway; 8 Centre for Clinical Research, Haukeland University Hospital, Bergen, Norway; 9 Department of Surgery, Haugesund Hospital, Haugesund, Norway; 10 Hormone Laboratory, Department of Medical Biochemistry and Pharmacology, Haukeland University Hospital, Bergen, Norway; 11 Department of Clinical Medicine, University of Bergen, Bergen, Norway; 12 Department of Medicine, Haukeland University Hospital, Bergen, Norway; 13 Regional Advisory Unit for Palliative Care, Department of Oncology, Oslo University Hospital, Oslo, Norway; 14 Department of Family Medicine and Community Health, Duke University School of Medicine, Durham, NC USA; 15 Department of Nursing and Health Promotion, Faculty of Health Sciences, Oslo Metropolitan University, Oslo, Norway

**Keywords:** Obesity, Obesity

## Abstract

**Background/objectives:**

There is limited long-term data comparing the outcomes of sleeve gastrectomy (SG) and Roux-en-Y gastric bypass (RYGB) for severe obesity, both with respect to body weight, quality of life (QOL) and comorbidities.

We aimed to determine 7-year trajectories of body mass index (BMI), QOL, obesity-related comorbidities, biomarkers of glucose and lipid metabolism, and early major complications after SG and RYGB.

**Subjects/methods:**

Patients scheduled for bariatric surgery at two Norwegian hospitals, preferentially performing either SG or RYGB, were included consecutively from September 2011 to February 2015.

Data was collected prospectively before and up to 7 years after surgery. Obesity-specific, generic and overall QOL were measured by the Impact of Weight on Quality of Life-Lite, Short-Form 36 and Cantril’s ladder, respectively. Comorbidities were assessed by clinical examination, registration of medication and analysis of glucose and lipid biomarkers. Outcomes were examined with linear mixed effect models and relative risk estimates.

**Results:**

Of 580 included patients, 543 (75% women, mean age 42.3 years, mean baseline BMI 43.0 kg/m^2^) were operated (376 SG and 167 RYGB). With 84.2% of participants evaluable after 5–7 years, model-based percent total weight-loss (%TWL) at 7 years was 23.4 after SG versus 27.3 after RYGB (difference 3.9%, *p* = 0.001). All levels of QOL improved similarly after the two surgical procedures but remained below reference data from the general population at all timepoints. Remission rates for type 2 diabetes, dyslipidemia, obstructive sleep-apnea and gastroesophageal reflux disease (GERD) as well as the rate of de novo GERD significantly favored RYGB. SG had fewer major early complications, but more minor and major late complications combined over follow-up.

**Conclusion:**

In routine health care, both SG and RYGB are safe procedures with significant long-term weight-loss, improvement of QOL and amelioration of comorbidities. Long-term weight-loss and remission rates of main obesity-related comorbidities were higher after RYGB.

## Introduction

Obesity affects 650 million people worldwide and is associated with a number of obesity-related diseases such as type 2 diabetes (T2D) and cardiovascular diseases, reduced life expectancy and lower quality of life (QOL) [1, 2]. Each 5 kg/m^2^ increase of body-mass index (BMI) above the normal range of 18–25 kg/m^2^ is associated with a 30% increase in overall mortality, and BMI above 40 kg/m^2^ may reduce life expectancy by 8–10 years [3]. Also for Norway there has been a steady increase in obesity and related comorbidities over the last decades [4].

Currently, bariatric surgery is the most effective treatment for severe obesity, defined as BMI above 40 kg/m^2^, or above 35 kg/m^2^ in the presence of obesity-related comorbidities with a suggested benefit also for lower BMI categories of 30–35 kg/m^2^ [5–7]. Roux-en-Y gastric bypass (RYGB) has been considered the gold standard bariatric procedure for decades. However, in recent years sleeve gastrectomy (SG) surpassed RYGB as the most frequently performed bariatric procedure, despite insufficient comparative data regarding long-term efficacy and safety [8, 9]. Early non-randomized studies showed no major differences between SG and RYGB in long-term weight loss, effect on comorbidities, or safety as measured by complication rates [10, 11].

Two randomized controlled trials (RCT), SLEEVEPASS and SM-BOSS, compared 5-year results after SG and RYGB in patients with severe obesity with no clinically relevant differences in weight loss or most other weight related outcomes [12, 13]. However, gastro-esophageal reflux disease (GERD), a condition both associated with obesity and a possible complication of bariatric surgery, appeared more prevalent after SG. Merged data from SLEEVEPASS and SM-BOSS revealed a small, but significantly greater weight loss after RYGB, along with higher remission rates of hypertension, dyslipidemia and GERD, but no difference in QOL improvements compared to SG [14]. Complication rates were lower after SG, suggesting a different balance between efficacy and adverse events. Meta-analyses did not find differences in long-term weight loss, QOL or T2D improvement, while control of dyslipidemia and hypertension was found to be either similar or to favor RYGB [15, 16].

Other RCTs have primarily studied the effect of bariatric surgery on weight-related comorbidities, especially T2D. The STAMPEDE trial demonstrated significantly better diabetes control in terms of freedom from antidiabetic medication 5 years after RYGB compared to SG in patients with obesity-related severe T2D [17]. Similarly, the recent OSEBERG trial revealed higher remission rates of T2D after RYGB compared to SG after one year [18].

Results from stringent RCTs may have limitations in terms of generalizability and applicability to clinical practice [19, 20]. To supplement data from RCTs, long-term observational studies are needed comparing the outcome after different bariatric procedures. Since 2009, SG and RYGB have been the most common bariatric procedures in Norway, with local variations between hospitals, some preferring SG and others RYGB. We therefore prospectively compared long-term outcomes after surgery in patients with severe obesity in a real-world clinical setting in Western Norway. To better capture the impact of bariatric surgery on patients’ QOL, we differentiated between narrow QOL concepts associated with changes of body weight, and broader aspects of QOL employing obesity-specific-, generic health-related and overall QOL questionnaires [21, 22].

Our primary objective was to determine trajectories of BMI and different levels of QOL after SG compared to RYGB. Secondary objectives were long-term changes in obesity-related comorbidities, glucose and lipid metabolism, and rates of early complications.

## Methods

The project “Bariatric Surgery on The West Coast of Norway” was conducted as a two-center observational study and approved by the Regional Committee for Medical and Health Research Ethics – Western Norway (2010/3287/REK, ClinicalTrials.gov: NCT01533142).

Two hospitals serving non-overlapping geographical regions participated. Voss Hospital (representing Bergen health region) has offered bariatric surgery since 2008 with currently about 200 procedures annually. At Haugesund Hospital (representing Fonna health region) bariatric surgery has been performed since 2007 with approximately 100 procedures annually. During the study period, the dominant method of surgery was SG at Voss Hospital and RYGB at Haugesund Hospital.

Patients scheduled for bariatric surgery were invited to participate in the study. Eligibility criteria were BMI ≥ 40 kg/m^2^ or ≥35 kg/m^2^ with obesity-related comorbidities, age 18 to 70 years, no alcohol or drug abuse, and no active psychosis. Written informed consent was obtained from all patients prior to inclusion. We collected demographic, clinical, biochemical, and QOL data using standardized checklists and validated questionnaires 2–3 months before surgery, and at routine outpatient visits 3 months, 1, 2, and 5 years postoperatively. Patients’ electronic hospital records were reviewed to complete data. Five-year data was supplemented with an electronically administered survey on average 7 years after surgery. At all timepoints, blood samples were obtained after overnight fasting and serum analyses performed according to the hospitals’ routine procedures. Analyses of insulin were performed at the Haukeland University Hospital (Bergen, Norway).

### Surgical procedures

Patients were allocated to SG or RYGB according to the preferred procedure at their respective hospital, but in a limited number of cases an individual decision as to the surgical procedure was allowed. Specified pre- and postoperative care was similar at both hospitals including prescription of a low-calorie diet (<1000 kcal per day) 3–4 weeks prior to surgery. Both surgical procedures were done laparoscopically. SG was performed with a gastric resection using a 32 French tube, starting 2–5 cm proximal to the pylorus and ending at the cardia, typically 0–1 cm from the angle of His. Due to updates on the surgical procedure during the study period, staple line reinforcement was performed in 99 patients and gastropexia in 131. Hiatal repair (*n* = 19) was performed when deemed medically indicated intra-operatively. RYGB was performed with a small gastric pouch, an antecolic end-to-side gastrojejunostomy, an alimentary limb of 100–150 cm, a side-to-side jejunostomy and a biliopancreatic limb of 40–60 cm. At the time of the study, mesenteric defects were not routinely closed (done in 5 cases). All operations were performed by experienced laparoscopist, allowing <10% of the procedures to be performed by novice professionals under supervision.

### Outcome definitions

Weight and obesity-related comorbidities were assessed according to international guidelines [23]. Weight loss was defined by percent total body weight loss (%TWL, weight change / initial weight * 100) and percentage excess BMI loss (%EBMIL, change in BMI / (initial BMI – 25) * 100). Baseline weight (in light clothing without shoes to the nearest 0.1 kilogram), height (in a standing position without shoes to the nearest 0.01 meters), and BMI were recorded at the first preoperative visit. Suboptimal weight loss was defined as %EBMIL below 50 or %TWL below 20 [24].

We obtained QOL measures at three levels: (1) obesity-specific QOL representing patients’ perception specifically related to their weight (2) generic health-related QOL representing broad domains of physical and mental health, and (3) Overall QOL, representing satisfaction with life as a whole [22].

For obesity-specific QOL, the validated Norwegian translation of The Impact of Weight on Quality of Life-Lite (IWQOL-Lite) questionnaire was applied, a measure of QOL related to weight. Five subscales measuring the impact of body weight on (1) physical functioning, (2) self-esteem, (3) sexual life, (4) public stress and (5) work life function were transformed to an overall total score from 0 to 100 with higher scores indicating better QOL [25, 26]. For comparison we used reference data from the US general population, including individuals in all BMI categories [27].

Generic health-related QOL was assessed using the validated Norwegian translation of Short-Form-36 (SF-36) [28]. The questionnaire encompasses 8 dimensions reflecting (1) physical functioning, (2) physical role functioning, (3) bodily pain, (4) general health, (5) vitality, (6) social functioning, (7) emotional role functioning and (8) mental health. Physical (PCS) and mental (MCS) composite scores were based on factor analysis with oblique rotation, and higher scores represent better QOL [29]. Reference values from the Norwegian general population in 2015 were available for comparison [30].

Overall QOL was captured using an adapted version of Cantril’s ladder, an overall measure of the patient’s subjective well-being in life [31] with one item, “All in all, how satisfied are you with your life at the moment?” and scores ranging from 0 (not at all satisfied) to 10 (highly satisfied). A score of 6 or more is labeled “high life satisfaction” and less than 6 “low life satisfaction”. For comparison we used reference data from the Norwegian general population including all BMI categories [32].

In patients with T2D at baseline, complete remission was defined as a glycated hemoglobin (HbA1c) value < 6.0% and fasting blood glucose (FBG) level <5.6 mmol/L, and partial remission was defined as HbA1c < 6.5% and FBG < 6.9 mmol/L, both without antidiabetic medication. Improvement was defined as a reduction in HbA1c and FBG not meeting criteria for remission or decrease in antidiabetic medication requirement. The index Homeostatic Model Assessment for Insulin Resistance (HOMA-IR) was calculated using the formula fasting insulin (in mU/L) * FBG (in mmol/L) / 22.5 and lower values indicate healthier glucose metabolism.

In patients with dyslipidemia at baseline, remission was defined by a level of low-density lipoprotein (LDL) cholesterol of <3.4 mmol/L without the need of medication. Cardiovascular risk was assessed by the total cholesterol/high-density lipoprotein (HDL) cholesterol ratio [23]. In patients with hypertension at baseline, a complete or partial remission was defined by a blood pressure not exceeding 120 mmHg/80 mmHg, or prehypertensive levels of 120–140 mmHg / 80–89 mmHg, respectively, both without the need of medication. Improvement was recorded in cases with a reduction in medication or significantly lower blood pressure on the same medication.

The presence of GERD, obstructive sleep-apnea (OSA), depression or anxiety at baseline was based on use of medication, or ventilation support (OSA). Resolution was defined as absence of symptoms and discontinuation of medication or ventilation support (GERD and OSA) or discontinuation of medication (depression and anxiety).

Early major postoperative complications within 30 days and late major complications were classified as Clavien-Dindo ≥3b [33]. Severe GERD, defined as symptoms not relieved by medication (as proposed in the BEST protocol [24]) and chronic abdominal pain for >3 months with a visual analogue scale of 6 or higher were recorded as minor late complications [24]. Patients who underwent a second bariatric operation during follow-up were excluded from subsequent analysis. Length of hospital stay was counted from day of operation to discharge from hospital to home, excluding intermittent days outside of hospital care.

Overall satisfaction with surgery was measured by the question: “How satisfied are you, all things considered, with the outcome after the bariatric operation?” with 4 response categories from “highly satisfied” to “not satisfied” [34].

### Statistical analysis

Categorical and continuous variables are presented as percentages, relative risk (RR) and mean values with standard deviations (SD) or 95% confidence intervals (CI). Groups of patients at defined timepoints were compared using chi-square and two sample *t* tests as appropriate. For comparisons with reference populations, we used one sample *t* tests for Cantril’s ladder and IWQOL-Lite, and two sample *t* tests for PCS and MCS. For continuous outcome variables, effect-sizes for differences of means were assessed by Cohen’s d and interpreted as follows: trivial (<0.2), small (0.2 to <0.5), moderate (0.5 to <0.8) or large (≥0.8) [35].

Changes over time in continuous variables were examined with linear mixed effect models (LMM). Models included all patients adjusted for sex, age at operation and BMI at baseline, surgery method and time from surgery as random factors. For HOMA-IR, only patients without T2D at baseline were entered. All models include interaction of time and surgery method. For assessment of comorbidities, data collection was not complete at all timepoints and we therefore merged data from 1 and 2 years and 5 and 7 years into two timepoints referred to as short- and long-term follow-up, respectively. In cases with sufficient data to assess comorbidities at both 1 and 2 years, we used only the former, and in cases with adequate data at both 5 and 7 years, we used the latter. Two-sided *p* values are reported without adjustments for multiple comparisons [36].

Data was analyzed with IBM SPSS (Statistics for Windows, Version 27.0. IBM Corp, Armonk, NY) and Stata SE (Stata Statistical Software: Release 15, StataCorp LLC, College Station, TX).

## Results

**Fig. 1:**
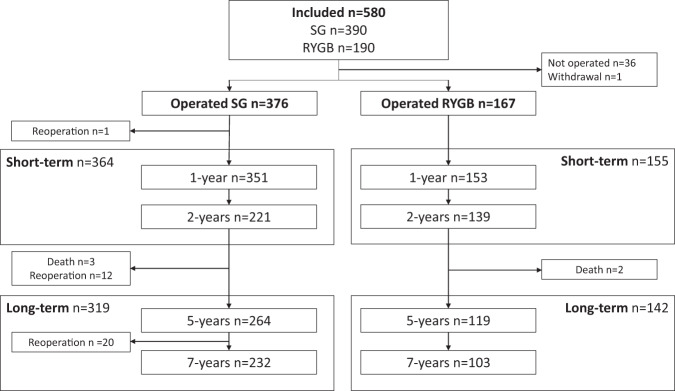
Flow-chart of included patients. Merged short- and long-term time points include patients with data available from 1 and/or 2 years or 5 and/or 7 years follow-up. RYGB Roux-en-Y gastric bypass, SG Sleeve gastrectomy, n number of patients with data registration at respective timepoint.

Between September 2011 and February 2015, 950 patients were scheduled for surgery at the two hospitals and 580 (61%) were enrolled (Fig. 1). Thirty-seven patients were excluded: 36 did not undergo surgery and one withdrew consent, for a total of 543 operated patients (376 SG and 167 RYGB). Voss hospital performed 350 SG and 10 RYGB procedures, while Haugesund hospital performed 157 RYGB and 26 SG procedures. One patient with a mesenterial vein thrombosis underwent a bowel resection within 30 days of surgery. Five patients died during follow-up (3 SG and 2 RYGB) from causes unrelated to surgery (2 presumed drug intoxications, 1 metastatic pulmonary cancer, 1 brain hemorrhage and 1 domestic accident). Twelve patients in the SG group underwent conversion to one-anastomosis gastric bypass (OAGB, *n* = 6) or RYGB (*n* = 6) between 24 and 60 months of follow-up, as did 20 patients (13 OAGB and 7 RYGB) between 60 and 84 months. These were all excluded from further follow-up at the time of the second operation.

At baseline there were no significant differences in sociodemographic or weight-related parameters between the groups, except more patients with higher education undergoing SG (Table 1).

**Table 1:** Baseline characteristics for all patients and by surgery method.

Characteristic^a^	All (*N* = 543)	Sleeve gastrectomy (*N* = 376)	Roux-en-Y gastric bypass (*N* = 167)	*P* value^b^
Age (years)	42.3 ± 11.3	42.6 ± 11.5	41.6 ± 10.9	0.34
Women	407/543 (75.0%)	282/376 (75%)	125/167 (74.9%)	0.97
Weight (kg)	124.7 ± 19.1	124.6 ± 19.1	125.0 ± 18.9	0.81
Body mass index (kg/m^2^)	43.0 ± 4.9	42.8 ± 5.3	43.1 ± 5.0	0.48
Body mass index ≥ 50 kg/m^2^	56/543 (10.3%)	38/376 (10.1)	18/167 (10.8%)	0.81
Married or cohabitants	244/343 (71.1%)	165/234 (70.5%)	79/109 (72.5%)	0.71
Higher education^**c**^	181/439 (41.2%)	132/281 (47.0%)	49/158 (31.0%)	**0.001**
Employed	297/541 (54.9%)	210/374 (56.0%)	87/167 (52.1%)	0.38
Type 2 diabetes	66/543 (12.2%)	52/376 (13.8%)	14/167 (8.4%)	0.07
Hypertension	138/541 (25.4%)	100/374 (26.6%)	38/167 (22.8%)	0.33
Dyslipidemia	67/541 (12.4%)	48/374 (12.8%)	19/167 (11.4%)	0.64
Obstructive sleep-apnea	61/541 (11.3%)	42/374 (11.2%)	19/167 (11.4%)	0.96
Anxiety	71/537 (13.2%)	50/371 (13.3%)	21/166 (12.6%)	0.79
Depression	120/541 (22.2%)	80/374 (21.3%)	40/167 (24.0%)	0.51
Gastroesophageal reflux disease	77/541 (14.2%)	48/374 (12.8%)	29/167 (17.4%)	0.16
Physical composite score	37.0 ± 9.2 (463)	36.8 ± 9.1 (317)	37.5 ± 9.4 (146)	0.50
Mental composite score	41.7 ± 10.7 (463)	41.2 ± 10.9 (317)	42.8 ± 10.5 (146)	0.15
Impact of weight on quality of life-lite total score	49.8 ± 20.6 (395)	49.3 ± 20.3 (278)	51.0 ± 21.4 (117)	0.47
Cantril’s ladder	5.1 ± 1.7 (140)	5.2 ± 1.7 (23)	5.0 ± 1.8 (117)	0.74

^a^Mean and standard deviation for continuous variables, number and percentages for categorical valuables.

^b^*P*-value for comparison of baseline characteristics between the surgical groups. *P*-values below 0.05 in bold.

^c^More than 13 years of school.

During follow-up we obtained short-term data from 364 patients after SG and 155 after RYGB. Similarly, long-term data was obtained from 319 and 142 after SG and RYGB, respectively (Fig. 1). Fifty-seven (15.2%) and 25 (15.0%) patients in the SG and RYGB groups were lost during follow-up. There were no major differences at baseline for patients with or without data at long-term follow-up, except for employment status in RYGB-treated and overall QOL in SG-treated patients (Supplementary Table 1).

### Weight loss

**Fig. 2:**
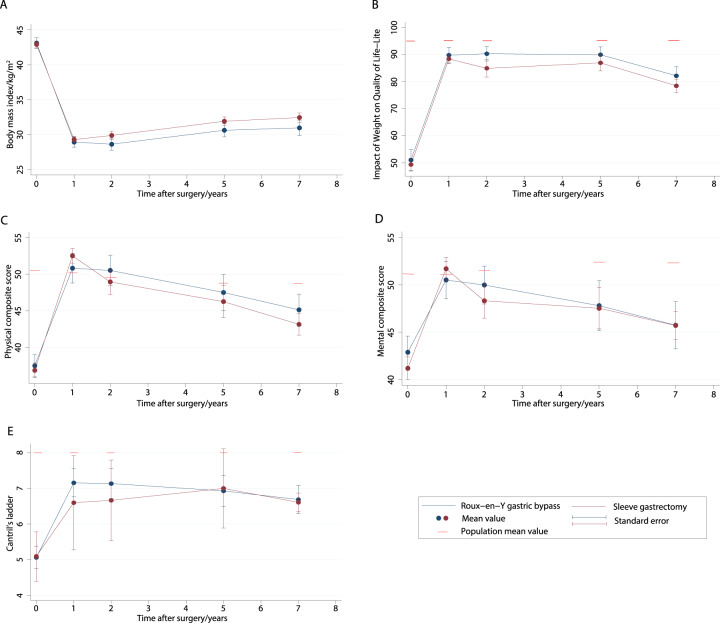
Seven-year trajectories of body weight and quality of life. Mean body mass index (**A**), Impact of weight on quality of life-Lite (**B**), physical composite score (**C**), mental composite score (**D**), and Cantril’s ladder (**E**) from preoperatively to 7 years after sleeve gastrectomy and Roux-en-Y gastric bypass. Sex and age adjusted population means for Impact of weight on quality of life, physical and mental composite scores and Cantril’s ladder for comparison (**B**, **C**, **D**, and **E**).

In both groups, there was significant weight loss, with most weight lost at 1–2 years after surgery and a gradual incline in body weight thereafter (Fig. 2 and Table 2). Weight loss was significantly different in the two surgery groups as indicated by highly significant p-values for the interaction term of time and surgery method included in the LMM. Specifically, the model-based mean %TWL at 7 years was 23.4 (95% CI 23.4, 24.5) for SG and 27.3 (25.8, 28.8) for RYGB (*p* = 0.001), with a between-group difference of 3.9%, and a small effect-size of 0.3. Similarly, %EBMIL at 7 years differed significantly, 57.9 (95% CI 55.4, 60.6) after SG versus 68.0 (64.2, 71.9) after RYGB (*p* < 0.001), corresponding to a between-group difference of 10.1% and a small effect-size of 0.4.

**Tabel 2:** Model-based means for continuous outcomes after Roux-en-Y gastric bypass and sleeve gastrectomy.

Outcome^a^	Type of surgery	Baseline	1 year	2 years	5 years	7 years	Linear mixed model with interaction
							*P* value
Weight (kg)/n	RYGB	124.4 (122.5, 126.3) / 167	83.1 (81.2, 85.1) / 153	83.0 (81.0, 85.0) / 139	89.3 (87.2, 91.4) / 119	89.8 (87.7, 92.0) / 103	**<0.001**^**b**^
	SG	124.5 (123.2, 125.7) / 376	84.6 (83.3, 85.9) / 351	87.5 (86.0, 88.9) / 221	92.6 (91.2, 94.0) / 264	95.0 (93.0, 95.6) / 232	0.938^c^
	Difference	0.1 (−2.2, 2.4)	1.34 (−1.0, 3.7)	4.2 (1.7, 6.7)	3.1 (0.6, 5.69)	4.3 (1.7, 6.9)	**<0.001**^**d**^
Body mass index (kg/m^2^)/*n*	RYGB	43.0 (42.5, 43.5) / 167	28.8 (28.2, 29.3) / 151	28.7 (28.1, 29.2) / 139	30.8 (30.2, 31.4) / 119	31.0 (30.4, 31.6) / 103	**<0.001**^**b**^
	SG	42.9 (42.5, 43.2) / 376	29.2 (28.8, 29.5) / 350	30.2 (29.7, 30.6) / 221	31.9 (31.5, 32.4) / 264	32.7 (32.3, 33.1) / 232	0.721^c^
	Difference	−0.1 (−0.7, 0.5)	0.4 (−0.2, 1.1)	1.5 (0.8, 2.2)	1.1 (0.4, 1.9)	1.7 (0.9, 2.5)	**<0.001**^**d**^
% Excess body mass index loss/*n*	RYGB		81.2 (77.7, 84.7) / 153	83.0 (79.4, 86.6) / 139	69.9 (66.2, 73.7) / 119	68.0 (64.2, 71.9) / 103	**<0.001**^**b**^
	SG		78.3 (76.0, 80.6) / 351	72.8 (70.2, 75.4) / 221	62.2 (59.7, 64.7) / 264	57.9 (55.4, 60.6) / 232	0.181^c^
	Difference		−2.9 (−7.1, 1.3)	−10.2 (−14.7, −5.7)	−7.7 (−11.2, −3.2)	−10.1 (−14.8, −5.4)	**<0.001**^**d**^
% Total weight loss/*n*	RYGB		32.8 (31.4, 34.2) / 154	33.1 (31.7, 34.5) / 139	27.9 (26.4, 29.4) / 119	27.3 (25.8, 28.8) / 103	**<0.001**^**b**^
	SG		31.6 (30.7, 32.6) /351	29.4 (28.4, 30.5) / 221	25.2 (24.2, 26.2) / 257	23.4 (22.4, 24.5) / 232	0.178^c^
	Difference		−1.1 (−2.8, 0.5)	−3.7 (−5.4, −1.9)	−2.7 (−4.5, −0.9)	−3.9 (−5.7, −2.0)	**0.001**^**d**^
Physcial composite score/*n*	RYGB	37.5 (35.9, 39.0) / 146	50.4 (48.5, 52.4) / 89	50.1 (48.2, 52.1) / 82	47.2 (45.1, 49.3) / 71	45.3 (43.5, 47.2) / 100	**<0.001**^**b**^
	SG	36.7 (35.6, 37.7) / 317	52.0 (50.9, 53.2) / 254	49.3 (47.9, 50.8) / 143	46.2 (44.5, 47.9) / 102	43.9 (42.7, 45.1) / 230	0.419^c^
	Difference	−0.8 (−2.7, 1.1)	1.6 (−0.6, 3.9)	−0.8 (−3.3, 1.7)	−1.0 (−3.7, 1.7)	−1.5 (−3.7, 0.7)	0.171^d^
Mental composite score/*n*	RYGB	43.1 (41.3, 44.8) / 146	50.1 (48.0, 52.2) / 89	49.2 (47.0, 51.4) / 82	47.2 (44.9, 49.5) / 71	45.8 (43.8, 47.8) / 101	**<0.001**^**b**^
	SG	41. (40.0, 42.3) / 317	51.2 (50.0, 52.5) / 254	48.9 (47.3, 50.2) / 143	46.9 (45.0, 48.8) / 102	45.9 (44.6, 47.2) / 230	0.075^c^
	Difference	−2.0 (−4.0, 0.1)	1.0 (−1.5, 3.5)	−0.5 (−3.2, 2.3)	−0.4 (−3.4, 2.6)	−0.2 (−2.5, 2.2)	0.241^d^
Impact of Weight on Quality of Life−Lite total score/*n*	RYGB	50.8 (47.7, 53.8) / 117	87.8 (83.9, 91.6) / 67	88.0 (84.1, 91.8) / 64	86.6 (82.7, 90.6) / 62	82.3 (79.0, 85.6) / 98	**<0.001**^**b**^
	SG	49.2 (47.3, 51.2) / 278	87.2 (84.9, 89.4) / 211	86.1 (83.3, 88.8) / 125	84.8 (81.7, 87.9) / 95	79.3 (77.1, 81.5) / 215	0.409^c^
	Difference	−1.5 (−5.2, 2.1)	−0.6 (−5.0, 3.8)	−1.9 (−6.6, 2.9)	−1.8 (−6.8, 3.2)	−3.0 (−6.9, 0.9)	0.918^d^
Cantril’s ladder/*n*	RYGB	5.1 (4.7, 5.4) / 117	7.0 (6.7, 7.4) / 82	7.1 (6.7, 7.5) / 80	6.7 (6.3, 7.1) / 71	6.7 (6.4, 7.1) / 101	**<0.001**^**b**^
	SG	5.1 (4.4, 5.8) / 23	6.5 (5.7, 7.3) / 16	6.4 (5.4, 7.4) / 9	6.67(5.8, 7.6) / 14	6.7 (6.5, 7.0) / 220	0.992^c^
	Difference	0.0 (−0.8, 0.8)	−0.5 (−1.4, 0.4)	−0.7 (−1.8, 0.4)	−0.0 (−1.0, 0.9)	−0.0 (−0.5, 0.4)	0.605^d^
Total cholesterol (mmol/L)/*n*	RYGB	4.63 (4.47, 4.80) / 126	4.33 (4.17, 4.49) / 146	4.38 (4.21, 4.54) / 127	4.59 (4.42, 4.77) / 104	5.00 (4.60, 5.40) / 14	**<0.001**^**b**^
	SG	4.95 (4.85, 5.06) / 326	5.18 (5.07, 5.29) / 310	4.99 (4.79, 5.18) / 65	5.16 (5.03, 5.28) / 186	5.22 (4.98, 5.56) / 42	**0.001**^**c**^
	Difference	0.32 (0.13, 0.51)	0.84 (0.65, 1.03)	0.59 (0.34, 0.85)	0.54 (0.32, 0.75)	0.12 (−0.33, 0.58)	**<0.001**^**d**^
Low Density Lipoprotein cholesterol (mmol/L)/*n*	RYGB	3.12 (2.96, 3.27) / 123	2.70 (2.52, 2.87) / 92	2.75 (2.56, 2.93) / 79	2.78 (2.60, 2.96) / 80	2.95 (2.57, 3.32) / 14	**<0.001**^**b**^
	SG	3.24 (3.15, 3.34) / 325	3.30 (3.20, 3.39) / 303	3.25 (2.99, 3.51) / 27	3.37 (3.25, 3.49) / 187	3.18 (2.97, 3.39) / 42	0.172^c^
	Difference	0.13 (−0.06, 0.31)	0.60 (0.40, 0.80)	0.50 (0.19, 0.82)	0.59 (0.37, 0.80)	0.23 (−0.19, 0.66)	**<0.001**^**d**^
High Density/*n* Lipoprotein cholesterol (mmol/L)/*n*	RYGB	0.99 (0.93, 1.04) / 123	1.31 (1.25, 1.37) / 92	1.41 (1.34, 1.47) / 79	1.45 (1.39, 1.52) / 80	1.68 (1.55, 1.81) / 14	**<0.001**^**b**^
	SG	1.06 (1.02, 1.09) / 326	1.57 (1.53, 1.60) / 303	1.55 (1.46, 1.64) / 27	1.62 (1.58, 1.66) / 181	1.67 (1.60, 1.75) / 42	**0.040**^**c**^
	Difference	0.07 (0.00, 0.13)	0.25 (0.18, 0.33)	0.13 (0.02, 0.24)	0.17 (0.09, 0.24)	0.00 (−0.14, 0.15)	**<0.001**^**d**^
Triglycerides (mmol/L)/*n*	RYGB	1.63 (1.47, 1.79) / 123	1.04 (0.86, 1.22) / 92	1.04 (0.85, 1.23) / 79	1.25 (1.06, 1.44) / 80	1.35 (0.94, 1.77) / 14	**<0.001**^**b**^
	SG	1.95 (1.85, 2.05) / 324	1.18 (1.08, 1.28) / 303	1.24 (0.99, 1.50) / 35	1.31 (1.19, 1.44) / 182	1.68 (1.45, 1.92) / 42	**0.001**^**c**^
	Difference	0.33 (0.14, 0.51)	0.14 (−0.07, 0.35)	0.20 (−0.12, 0.52)	0.06 (−0.16, 0.29)	0.33 (−0.15, 0.81)	0.224^d^
HbA1C (%)/*n*	RYGB	5.8 (5.7, 6.0) / 97	5.4 (5.2, 5.6) / 75	5.5 (5.3, 5.7) / 81	5.6 (5.4, 5.8) / 73	5.7 (5.40, 6.0) / 23	**<0.001**^**b**^
	SG	6.0 (5.9, 6.1) / 310	5.5 (5.4, 5.6) / 237	5.4 (5.2, 5.5) / 90	5.6 (5.5, 5.7) / 193	5.7 (5.6, 5.9) / 72	**0.034**^**c**^
	Difference	0.2 (0.0, 0.4)	0.1 (−0.1, 0.3)	−0.1 (−0.4, 0.1)	−0.0 (−0.2, 0.2)	0.1 (−0.3, 0.4)	0.089^d^
Fasting blood glucose (mmol/L)/*n*	RYGB	6.0 (5.8, 6.3) / 151	4.9 (4.7, 5.2) / 146	5.0 (4.7, 5.3) / 127	5.3 (5.0, 5.6) / 106	5.4 (4.8, 6.1) / 18	**<0.001**^**b**^
	SG	6.6 (6.5, 6.8) / 369	5.1 (5.0, 5.3) / 313	5.2 (4.9, 5.5) / 96	5.5 (5.3, 5.7) / 205	5.6 (5.3, 6.0) / 61	**<0.001**^**c**^
	Difference	0.6 (0.3, 0.9)	0.2 (−0.1, 0.5)	0.2 (−0.2, 0.6)	0.1 (−0.2, 0.5)	0.2 (−0.5, 1.0)	0.122^d^
Homeostatic Model Assessment for Insulin Resistance^e^	RYGB	3.98 (3.52, 4.43) / 102	0.92 (0.43, 1.42) / 87	1.36 (0.82, 1.90) / 74	1.90 (1.38, 2.42) / 78	3.51 (2.17, 4.86) / 12	**<0.001**^**b**^
	SG	3.06 (2.76, 3.36) / 250	1.31 (1.04, 1.57) / 302	1.66 (0.84, 2.49) / 42	2.50 (2.15, 2.85) / 185	3.18 (2.45, 3.92) / 40	**<0.001**^**c**^
	Difference	−0.92 (−1.46, −0.37)	0.38 (−0.18, 0.95)	0.30 (−0.68, 1.28)	0.60 (−0.02, 1.22)	−0.33 (−1.86, 1.19)	**0.002**^**d**^
Cardiovascular risk	RYGB	4.9 (4.7, 5.1) / 123	3.4 (3.2, 3.7) / 92	3.2 (3.0, 3.5) / 79	3.3 (3.1, 3.5) / 80	3.1 (2.6, 3.6) / 14	**<0.001**^**b**^
	SG	4.9 (4.8, 5.1) / 326	3.5 (3.4, 3.6) / 303	3.4 (3.0, 3.7) / 26	3.3 (3.1, 3.5) / 181	3.5 (3.2, 3.7) / 42	0.789^c^
	Difference	0.0 (−0.2, 0.3)	0.1 (−0.2, 0.3)	0.1 (−0.3, 0.6)	0.1 (−0.2, 0.4)	0.4 (−0.2, 0.9)	0.840^d^

All values except ^e^ are predicted means (95% confidence intervals) from linear mixed model for all pateints with values recorded (*n*), adjusted for sex, age at operation and baseline body mass index, surgery method and time from surgery as random factors. All models include interaction of time and surgery method.

^a^Values in parentheses are 95 percent confidence intervals.

^b^Main effect of time, *p* values below 0.05 (bold) indicate significant change with time after surgery.

^c^Main effect of type of operation at baseline, *p* values below 0.05 (bold) indicate imbalance between groups at baseline.

^d^Main effect of interaction of operation and time, *p* values below 0.05 (bold) indicate significant effect of type of operation over time.

^e^Model based means for patients without diabetes at baseline.

Suboptimal weight loss at long-term follow-up (%EBMIL < 50) was seen in 118 of 319 (37.0%) patients after SG versus 34 of 142 (23.9%) after RYGB (*p* = 0.006). Percent TWL < 20 was documented in 112 of 319 (35.1%) patients after SG versus 29 of 142 (20.4%) after RYGB (*p* = 0.002).

### Long-term QOL outcomes

There were significant improvements in obesity-specific, generic and overall QOL after both SG and RYGB, with highest scores in all levels obtained at 1–2 years and a gradual decline thereafter (Fig. 2 and Table 2). There was no difference between patients operated with either SG or RYGB in QOL as indicated by the non-significant *p* values for the interaction term included in the LMM.

The mean differences in IWQOL-Lite, PCS, MCS, and Cantril’s ladder from baseline to 7 years were 31.2 (95%CI 28.6, 33.9), 7.5 (6.1, 8.9), 3.9 (2.3, 5.6) and 1.6 (1.2, 2.1) for all patients together, with effect-sizes of 1.5 (large), 0.6 (moderate), 0.3 (small) and 0.8 (moderate), respectively.

Mean scores for all levels of QOL were significantly lower than population means at all timepoints (Supplementary Table 2). The difference from reference means was smaller at 7 years follow-up compared to baseline for all levels, and effect-sizes for the difference decreased from 0.8 to 2.2 at baseline to 0.3–0.8 at 7 years. The improvement relative to reference means, expressed as the difference in effect-size from baseline to 7 years follow-up, was highest for IWQOL-Lite (reduction in effect-size from 2.0 to 0.8 and 2.2 to 0.7 after SG and RYGB, respectively). The smallest improvement was seen for MCS (reduction from 0.8 to 0.6 and 0.8 to 0.5 after SG and RYGB, respectively).

### Metabolic changes and comorbidities associated with severe obesity

Overall, patients in both groups showed significant improvements in blood biomarkers of glucose and lipid metabolism and improvements in prevalence of main obesity-related comorbidities (Tables 2 and 3).

**Table 3:** Early postoperative complications and short-term (1–2 years) and long-term (5–7 years) changes in obesity-related comorbidities.

Outcome^a^	All patients	Sleeve gastrectomy	Roux-en-Y gastric bypass	Relative risk^b^ (95% CI)	*P* value^c^
Early postoperative complications^**d**^					
Major events^**e**^	18/543 (3.3%)	8/376 (2.1%)	10/167 (6.0%)	0.36 (0.14, 0.89)	**0.026**
All early adverse events	48/543 (8.8%)	27/376 (7.2%)	21/167 (12.6%)	0.53 (0.33, 0.98)	**0.042**
Late substantial complications					
Major events^e^	39/464 (8.4%)	31/322 (9.6%)	8/142 (5.6%)	1.71 (0.81, 3.62)	0.162
All late adverse events^f^	124/464 (26.7%)	99/322 (30.7%)	25/142 (17.6%)	1.75 (1.18, 2.58)	**0.005**
Type 2 diabetes^g^					
Complete remission _short-term_	28/63 (44.4%)	22/50 (44.0%)	6/13 (46.2%)	0.95 (0.49, 1.85)	0.888
≥partial remission _short-term_	33/63 (52.4%)	27/50 (54.0%)	6/13 (46.2%)	1.17 (0.62, 2.22)	0.631
≥improved _short-term_	53/65 (81.5%)	42/51 (82.4%)	11/14 (78.6%)	1.05 (0.78, 1.42)	0.760
Complete remission _long-term_	6/47 (12.8%)	2/36 (5.6%)	4/11 (36.4%)	0.15 (0.03, 0.73)	**0.018**
≥partial remission _long-term_	14/47 (29.8%)	8/36 (22.2%)	6/11 (54.5%)	0.41 (0.18, 0.92)	**0.031**
≥improved _long-term_	30/54 (55.6%)	22/42 (52.4%)	8/12 (66.7%)	0.86 (0.46, 1.62)	0.640
de novo^h^_short-term_	13/454 (2.9%)	7/311 (2.3%)	6/143 (4.2%)	0.54 (0.18, 1.57)	0.255
de novo _long-term_	14/406 (3.4%)	10/276 (3.6%)	4/130 (3.1%)	1.18 (0.38, 3.68)	0.779
Relapse^i^_long-term_	16/38 (42.1%)	14/30 (46.7%)	2/8 (25.0%)	1.87 (0.53, 6.58)	0.332
Hypertension					
Complete remission _short-term_	10/116 (8.6%)	6/86 (7.0%)	4/30 (13.3%)	0.52 (0.16, 1.73)	0.288
≥partial remission _short-term_	38/102 (37.3%)	27/77 (35.1%)	11/25 (44.0%)	0.80 (0.47, 1.36)	0.407
≥improved _short-term_	81/119 (68.1%)	59/87 (67.8%)	22/32 (68.8%)	0.99 (0.75, 1.30)	0.922
Complete remission _long-term_	2/93 (2.2%)	0/69 (0.0%)	2/24 (8.3%)	0.07 (0.00, 1.44)	0.085
≥partial remission _long-term_	17/93 (18.3%)	11/69 (15.9%)	6/24 (25.0%)	0.64 (0.26, 1.54)	0.316
≥improved _long-term_	52/116 (44.8%)	36/84 (42.9%)	16/32 (50.0%)	0.86 (0.56, 1.31)	0.710
Dyslipidemia					
Remission _short-term_	17/53 (32.1%)	11/38 (28.9%)	6/15 (40.0%)	0.72 (0.33, 1.60)	0.425
Remission _long-term_	10/45 (22.2%)	3/31 (9.7%)	7/14 (50.0%)	0.19 (0.06, 0.64)	**0.007**
Gastro-esophageal reflux disease					
Remission _short-term_	12/56 (21.4%)	3/37 (8.1%)	9/19 (47.4%)	0.17 (0.05, 0.56)	**0.004**
Remission _long-term_	14/61 (23.0%)	2/37 (5.4%)	12/24 (50.0%)	0.11 (0.03, 0.44)	**0.002**
de novo^h^_short-term_	80/404 (19.8%)	73/276 (26.4%)	7/128 (5.5%)	4.84 (2.29,10.20)	**<0.001**
de novo _long-term_	102/362 (28.2%)	97/256 (37.9%)	5/106 (4.7%)	8.03 (3.37,19.17)	**<0.001**
Obstructive sleep-apnea					
Resolution _short-term_	46/57 (80.7%)	29/39 (74.4%)	17/18 (94.4%)	0.79 (0.63, 0.98)	**0.030**
Resolution _long-term_	31/50 (62.0%)	20/36 (55.6%)	11/14 (78.6%)	0.71 (0.47, 1.06)	0.090
Depression					
Resolution _short-term_	46/110 (41.8%)	37/76 (48.7%)	9/34 (26.5%)	1.84 (1.00, 3.37)	**0.049**
Resolution _long-term_	56/100 (56.0%)	38/68 (55.9%)	18/32 (56.3%)	0.99 (0.69, 1.44)	0.972
Anxiety					
Resolution _short-term_	22/56 (39.3%)	16/42 (38.1%)	6/14 (42.9%)	0.89 (0.43, 1.82)	0.748
Resolution _long-term_	39/59 (66.1%)	28/42 (66.7%)	11/17 (64.7%)	1.03 (0.68, 1.55)	0.887

^a^Number and percentages for categorical values.

^b^95% confidence (CI) interval in brackets.

^c^*P* value for relative risk between surgery groups. *P* values below 0.05 in bold.

^d^Within first 30 postoperative days.

^e^Clavien-Dindo 3b or higher.

^f^Including severe GERD defined as persisting symptoms not controlled by medication and chronic pain >3 months with visual analogue scale 6 or higher.

^g^Complete remission of type 2 diabetes was defined as HbA1c value <6.0% and fasting glucose level <5.6 mmol/L, and partial remission was defined as HbA1c < 6.5% and fasting glucose <6.9 mmol/L, both without antidiabetic medication.

^h^de novo defined as new onset of treatment for a comorbidity, e.g., type 2 diabetes requiring antidiabetic medication postoperatively.

^i^Relapse defined as re-initiation of treatment for a comorbidity, e.g., restart of antidiabetic medication after initial remission.

Long-term complete or partial remission rates in patients with T2D at baseline were significantly higher after RYGB, with RRs of 0.15 (*p* = 0.018) and 0.41 (*p* = 0.031), respectively. In non-diabetic patients at baseline, there was a significantly greater improvement in insulin resistance, assessed as HOMA-IR, after RYGB compared to SG (*p* = 0.002).

Long-term remission of dyslipidemia was significantly more common after RYGB than SG (RR 0.19, *p* = 0.007). RYGB was also associated with a significantly greater reduction in total and LDL cholesterol compared to SG (*p* < 0.001 for both). This did not translate into significant differences in the total/HDL cholesterol ratio, due to significantly higher levels of HDL cholesterol in patients after SG.

Remission of GERD was significantly more common after RYGB than SG at short- and long-term follow-up with RRs of 0.17 and 0.11 (*p* = 0.004 and 0.002), respectively. The occurrence of de novo GERD was 5 and 8 times more common after SG than RYGB at short- and long-term follow-up, respectively (*p* < 0.001).

### Adverse outcomes and satisfaction

Overall, 48 and 124 patients experienced early and/or late postoperative complications, respectively (Supplementary Table 3). Early complications of any severity and those classified as major were significantly more common after RYGB than SG (Table 3). Late complications of any severity were significantly more common after SG, but there was no difference in the rate of late major complications. Mean length of hospital stay for early major complications after SG was 50.0 (95% CI 20.2, 79.8) days and 15.5 (8.1, 22.9) after RYGB (*p* = 0.010), but did not differ comparing complications of any severity.

At long-term follow-up, 148 out of 225 patients after SG (65.8%) reported to be satisfied or highly satisfied with the treatment outcome, compared to 93 out of 124 RYGB patients (75%) (*p* = 0.292).

## Discussion

We compared 7-year outcomes after SG and RYGB for severe obesity under real-world conditions at two Norwegian hospitals. With high follow-up rates, the groups were balanced for important baseline characteristics with the exception of educational level. The hospital committed to RYGB performed a lower number of operations annually compared to the hospital conducting SG. With these limitations, short-term weight loss was considerable and similar after both procedures, but with a gradual weight regain from 1 to 2 years being more prominent after SG. QOL, assessed at obesity-specific, generic and overall levels, was similar in both groups at all timepoints. Long-term outcome for T2D, dyslipidemia, OSA and GERD favored RYGB, whereas early complications were less frequent after SG, but required longer in hospital stay.

At 7-year follow-up, the differences in modeled mean %TWL and %EBMIL were 3.9 and 10.1, respectively, both in favor of RYGB but with small effect-sizes. These numbers are close to the thresholds for clinically relevant differences of ±5% TWL and ±9% EWL defined in other studies [12, 13, 24]. Similarly, 7-year results from the SLEEVEPASS study showed a statistically significant difference in %EWL of 8.7 in favor of RYGB [37]. Two other comparable RCTs, the SM-BOSS and a smaller French study, also reported differences favoring RYGB at 5 years in terms of %EBMIL, %TWL or %EWL [13, 38]. A large unmatched registry study for the United States reported 6.2–8.1% higher TWL for RYGB compared to SG [39]. Together, accepting these thresholds for equivalence, our real-world results support the conclusion that SG and RYGB yield comparable clinical benefit in terms of weight loss, tending to favor RYGB in the long term. The ongoing Swedish BEST study specifies a non-inferiority margin of 5% difference in %TWL to balance the increased complication rate after RYGB compared to SG, also in accordance with our results [24].

Remission of GERD was seen more frequently at short- and long-term follow-up in the RYGB group, and de novo GERD occurred more commonly at long-term follow-up after SG. Similar results are seen in the most recent RCTs and reviews, suggesting RYGB to be the procedure of choice for patients with preexisting GERD [12, 13, 38, 40]. During the recruitment period of our study, there was no apparent selection of RYGB over SG evidenced by the similar prevalence of GERD at baseline in the two groups, and modifications of the SG procedure with gastropexia or hiatal repair were performed in a minority of cases only. Whether these technical adaptations of the SG procedure result in better control of reflux is still debated [41–43].

Despite only limited differences in weight loss, long-term T2D complete and partial remissions were more common after RYGB compared to SG. Regarding T2D prevention, using HOMA-IR as a measure of insulin resistance, non-diabetic patients at baseline showed higher long-term improvements after RYGB compared to SG. Notably, at short-term follow-up, HOMA-IR improved similarly after SG and RYGB, but the effect appeared to wane over time in the SG group. The SLEEVEPASS and SM-BOSS studies included 42 and 24.9% patients with T2D at baseline, respectively, and showed no difference in neither T2D remission nor biomarkers of glycemic control at 5-year follow up [13]. On the other hand, 5-year results of the STAMPEDE trial and 1-year results from the OSEBERG study together suggest better glycemic control after RYGB [17, 18]. Whether improved glycemic control after RYGB is a consequence of slightly better weight control seen in several major studies, or a direct effect of altered gut physiology, is currently an important research topic. A recent meta-analysis showed elevated levels of circulating bile acids after RYGB, but not after SG, possibly due to the anatomical construction of a biliopancreatic limb in RYGB [44]. Circulating bile acid concentrations were found to be inversely related to HOMA-IR [45].

Both the remission rate of dyslipidemia and reduction of total and LDL cholesterol levels were significantly better after RYGB. These findings are in accordance with 5-year results from the SLEEVEPASS study [12]. The SM BOSS study also reported significantly lower levels of LDL cholesterol after RYGB, but total cholesterol and remission rates of dyslipidemia only showed a trend in favor of RYGB [13]. LDL-cholesterol is recommended as the primary lipid analysis for screening, diagnosis, and management of dyslipidemia, and lowering of LDL is clinically meaningful since it reduces the risk of atherosclerotic cardiovascular disease [49]. However, the impact of surgery may be more complex, as we reported higher levels of HDL cholesterol at several timepoints after SG. Cardiovascular risk assessed as the ratio of total/HDL cholesterol improved similarly after both surgical procedures.

We found SG to be associated with fewer complications than RYGB within 30 days of surgery, corresponding to findings in SLEEVEPASS and SM-BOSS [12, 13]. The relative risk of early major (0.36) or any (0.53) complications are within the superiority margin regarding safety, favoring SG, defined by the Swedish BEST study [24]. However, the lower risk of major complications after SG may be offset by the significantly longer hospital stay following leaks after SG. With different definitions used, our rate of major early complications after RYGB seems comparable to the SM-BOSS and SLEEVEPASS trials, but higher than described by the Scandinavian Obesity Surgery Registry [12, 13, 46, 47]. Both socioeconomic factors, hospital volumes and surgeons’ experience have been described as determinants of complications rates following obesity surgery [46, 48]. Of note, in our study RYGB was performed in the hospital with the lowest volume of bariatric procedures, and on patients with a lower educational level compared to SG treated patients. The risk of any complication of Clavien-Dindo ≥3b within 5 years from surgery was 18.4% for the SM-BOSS and SLEEVEPASS studies combined, less favorable than the rate of any major complication of 8.4% in our study [14]. Of interest, we had only 3 cases of internal herniation requiring reoperation after RYGB, a major contributor to late complications in other studies.

QOL assessed at three different levels showed considerable improvements at 1–2 years and a gradual decline thereafter yielding bi-phasic patterns of parallel changes in BMI and QOL, as previously reported in RCTs and in our observational study after SG [13, 21, 38]. To our knowledge, a comprehensive assessment of QOL at different levels has not been done in other comparative trials of bariatric procedures. Despite higher weight loss and higher remission rates of major comorbidities after RYGB, neither the RCTs nor our study demonstrate any significant difference of these surgical methods on improvement of QOL. It is tempting to speculate that this discrepancy may in part be explained by differences in outcomes other than weight loss, such as adverse effects, or too few patients in each group to detect a significant difference (type 2 error). Furthermore, improvements in QOL from baseline to 7 years expressed in terms of effect-size appeared highest for the measures most closely related to weight as a physical phenomenon, i.e. the PCS of SF-36, and obesity-specific QOL. Mental components of SF-36, summarized in MCS, and overall QOL benefited less from bariatric surgery, similar to previous findings from our and other groups [21, 49]. QOL in many operated patients seems to remain at levels below the general population, particularly the mental dimension. This raises concerns that psychological needs of patients with obesity are not adequately addressed by surgery and routine follow-up programs [21, 49–51].

Our comprehensive QOL data may allow for a more in-depth analysis of separated domains from the different instruments and more detailed investigations of factors associated with improvements in QOL after surgery, such as complications, presence of depression and anxiety. Thus, possible predictors of long-term QOL will be explored in future studies.

### Strengths and limitations

Our study was a non-randomized comparison and therefore lacks the high internal validity of an RCT. Administrative short-comings in informing all potential participants represent the main reason for incomplete inclusion. Still, prospective inclusion at two hospitals with low to intermediate annual operation volumes, comparable capture areas and close adherence to one of two different surgical procedures, allowed for large and similar groups at baseline. Pragmatic in nature, follow-up and assessment of outcomes are not complete at all timepoints, but attrition is still low at long-term follow-up. Furthermore, we report broad validated measures of QOL covering obesity-specific, generic and overall domains relative to general population scores.

## Conclusion

Under routine conditions, both SG and RYGB are safe procedures for patients with severe obesity resulting in significant long-term weight loss, improvement of QOL and amelioration of comorbidities. RYGB may still be the yardstick by which to compare other procedures.

## Supplementary information

Supplementary table 1, 2 and 3
