# Supplementary material for: Seven-year trajectories of body weight, quality of life and comorbidities following Roux-en-Y gastric bypass and sleeve gastrectomy
Source: Int J Obes (Lond). 2022 Jan 1;46(4):739–49. doi: 10.1038/s41366-021-01028-5 (PMC8960397; doi:10.1038/s41366-021-01028-5)
Supplement: Supplementary file 1 — Supplementary table 1, 2 and 3 [file 41366_2021_1028_MOESM1_ESM.docx]

**Supplementary table 1. Baseline characteristics by surgical procedure and attendance at long-term follow-up**

|  | **Patients attending long-term follow-up** | | **Patients lost to follow-up** | | **P-value^b^** | |  |  |  |  |  |
| --- | --- | --- | --- | --- | --- | --- | --- | --- | --- | --- | --- |
|  | **N = 457 (84.2%)** | | **N = 86 (15.8%)** | |  |  |  |  |  |  |  |
| **Characteristic^a^** | **SG** | **RYGB** | **SG** | **RYGB** | **SG** | **RYGB** |  |  |  |  |  |
| Age at operation (years) | 42.6 ± 11.6 (319) | 42.1 ± 10.6 (142) | 42.5 ± 10.9 (57) | 38.5 ± 12.3 (25) | 0.98 | 0.13 |  |  |  |  |  |
| Women | 242/319 (76.2%) | 109/142 (76.8%) | 39/57 (68.4%) | 16/25 (64.0%) | 0.25 | 0.21 |  |  |  |  |  |
| Weight (kg) | 124.6 ± 19.1 (319) | 123.9 ± 18.5 (142) | 124.6 ± 19.8 (57) | 131.4 ± 20.4 (25) | 0.99 | 0.07 |  |  |  |  |  |
| Body mass index(kg/m^2^) | 42.9 ± 5.0 (319) | 42.9 ± 5.0 (142) | 42.9 ± 4.6 (57) | 44.2 ± 4.5 (25) | 0.93 | 0.24 |  |  |  |  |  |
| Body mass index ≥ 50 kg/m^2^ | 32/319 (10.0%) | 15/142 (10.6%) | 6/57 (10.5%) | 3/25 (12.0%) | 1.00 | 1.00 |  |  |  |  |  |
| Married or cohabitant | 152/211 (72.0%) | 74/101 (73.3%) | 13/23 (56.5%) | 5/8 (62.5%) | 0.15 | 0.68 |  |  |  |  |  |
| Higher education**^c^** | 111/242 (45.9%) | 46/136 (33.8%) | 21/39 (53.8%) | 3/22 (13.6%) | 0.39 | 0.08 |  |  |  |  |  |
| Employed | 178/317 (56.2%) | 80/142 (56.3%) | 32/57 (56.1%) | 7/25 (28.0%) | 1.00 | **0.01** |  |  |  |  |  |
| Type 2 diabetes | 42/319 (13.2%) | 12/142 (8.5%) | 10/57 (17.5%) | 2/25 (8.0%) | 0.41 | 1.00 |  |  |  |  |  |
| Hypertension | 84/318 (26.4%) | 32/142 (22.5%) | 16/57 (28.1%) | 6/25 (24.0%) | 0.87 | 1.00 |  |  |  |  |  |
| Dyslipidemia | 40/318 (12.6%) | 18/142 (12.7%) | 9/57 (15.8%) | 1/25 (4.0%) | 0.52 | 0.31 |  |  |  |  |  |
| Obstructive sleep apnea | 36/317 (11.4%) | 14/142 (9.9%) | 6/57 (10.5%) | 5/25 (20.0%) | 1.00 | 0.17 |  |  |  |  |  |
| Anxiety | 44/317 (13.9%) | 17/142 (12.0%) | 6/54 (11.1%) | 4/24 (16.7%) | 0.67 | 0.74 |  |  |  |  |  |
| Depression | 68/318 (21.4%) | 33/142 (23.2%) | 12/57 (21.1%) | 7/25 (28.0%) | 1.00 | 0.62 |  |  |  |  |  |
| Gastro-espohageal reflux disease | 43/318 (13.5%) | 26/142 (18.3%) | 5/57 (8.8%) | 3/25 (12.0%) | 0.39 | 0.57 |  |  |  |  |  |
| Physical composite score | 36.8 ± 9.0 (268) | 37.8 ± 9.2 (128) | 37.2 ± 9.8 (49) | 35.2 ± 10.9 (18) | 0.76 | 0.28 |  |  |  |  |  |
| Mental composite score | 41.3 ± 11.1 (268) | 43.3 ± 10.2 (128) | 42.2 ± 9.4 (49) | 39.0 ± 11.9 (18) | 0.96 | 0.10 |  |  |  |  |  |
| Impact of Weight on Quality of Life-Lite Total Score | 48.7 ± 20.2 (238) | 51.0 ± 21.8 (105) | 53.3 ± 21.0 (40) | 50.8 ± 18.7 (12) | 0.18 | 0.97 |  |  |  |  |  |
| Cantril's ladder | 5.5 ± 1.5 (19) | 5.2 ± 1.8 (103) | 3.5 ± 1.3 (4) | 4.2 ± 1.6 (14) | **0.02** | 0.06 |  |  |  |  |  |

SG, Sleeve gastrectomy; RYGB, Roux-en-Y gastric bypass

^a^Mean and standard deviation for continuous variables, number and percentages for categorical valuables.

^b^P-value for comparison of patients attending or lost at long-term follow-up by surgery method. P-values below 0.05 in bold. ^c^More than 13 years of education.

**Supplementary table 2. Differences from population mean scores for quality of life at baseline and at 7 years**

|  | **Physical composite score** | | **Mental composite score** | | **Impact of Weight on**  **Quality of Life-Lite Total Score** | | **Cantril’s ladder** | |
| --- | --- | --- | --- | --- | --- | --- | --- | --- |
|  | Mean difference/CI | p-value/  Cohen’s d | Mean difference/CI | p-value/  Cohen’s d | Mean difference/CI | p-value/  Cohen’s d | Mean difference/CI | p-value/  Cohen’s d |
| **Sleeve gastrectomy** | | | | | | | | |
| Baseline | -13.8 (-14.8, -12.8) | < 0.001  1.5 | -10.0 (-11.2, -8.8) | < 0.001  0.8 | -45.4 (-47.8, -43.0) | < 0.001  2.2 | -2.9 (-3.6, -2.2) | < 0.001  1.7 |
| 7 years | -5.7 (-7.1, -4.3) | < 0.001  0.5 | -6.8 (-8.3, -5.4) | < 0.001  0.6 | -16.3 (-18.7, -13.8) | < 0.001  0.8 | -1.4 (-1.7, -1.1) | < 0.001  0.7 |
| **Gastric bypass** | | | | | | | | |
| Baseline | -13.1 (-14.7, -11.6) | < 0.001  1.5 | -8.3 (-10.0, -6.6) | < 0.00  0.8 | -43.7 (-47.6, -39.8) | < 0.001  2.0 | -2.9 (-3.3, -2.6) | < 0.001  1.8 |
| 7 years | -2.8 (-4.7, -0.8) | 0.006  0.3 | -5.9 (-8.1, -3.8) | < 0.001  0.5 | -12.5 (-15.9, -9.2) | < 0.001  0.7 | -1.3 (-1.7, -0.9) | < 0.001  0.6 |

CI, 95% confidence intervals

**Supplementary table 3. Early and late complications of surgery**

| **Type of complication^a^** | **Sleeve gastrectomy (N=376)** | | **Roux-en-Y gastric bypass (N=167)** | |
| --- | --- | --- | --- | --- |
| **Minor** | **Early** | **Late** | **Early** | **Late** |
| Infections treated with antibiotics^b^ | 7 |  | 5 |  |
| Bleeding | 10 |  | 3 |  |
| Stenosis | 1 |  |  |  |
| Ulcer |  |  | 2 |  |
| Other | 1 |  | 1 |  |
| Severe GERD^c^ |  | 44 |  | 5 |
| Chronic abdominal pain^d^ |  | 24 |  | 12 |
| **Major** |  |  |  |  |
| Leak | 4 | 1 | 5 |  |
| Abscess | 2 |  |  |  |
| Stenosis |  | 2 | 1 | 2 |
| Internal herniation |  |  |  | 3 |
| Bowel perforation |  |  | 1 |  |
| Omentum necrosis | 1 |  |  |  |
| Mesenterial vein thrombosis | 1 |  |  |  |
| Ulcer |  |  |  | 3 |
| Revisional surgery for GERD |  | 26 |  |  |
| Incisional hernia |  | 2 | 2 |  |
| Bleeding |  |  | 1 |  |
| **Total** | **27** | **99** | **21** | **25** |

^a^Number of patients with complication

^b^Includes pneumonia (n=3), fever of unknown origin (n=3), wound infection (n=1) and urinary tract infection (n=1)

^c^ GERD symptoms that cannot be controlled by medication, not operated

^d^Pain (> 3 months) with visual analogue scale 6 or higher
